# Circulating proteasome activity following mild head injury in children

**DOI:** 10.1007/s00381-014-2409-4

**Published:** 2014-04-04

**Authors:** Marzena Tylicka, Ewa Matuszczak, Wojciech Dębek, Adam Hermanowicz, Halina Ostrowska

**Affiliations:** 1 Department of Pediatric Surgery, Medical University of Białystok, 15-274 Białystok, Poland; 2 Department of Biophysics, Medical University of Białystok, Mickiewicza 2A, 15-089 Białystok, Poland

**Keywords:** Mild head injury, Concussion, Head injury, Children, Circulating proteasome activity, Plasma

## Abstract

**Purpose:**

The aim of the study is to characterize changes in circulating proteasome (c-proteasome) activity following mild traumatic brain injury in children.

**Methods:**

Fifty children managed at the Department of Pediatric Surgery because of concussion—mild head injury was randomly included into the study. The children were aged 11 months to 17 years (median = 10.07 + −1.91 years). Plasma proteasome activity was assessed using Suc-Leu-Leu-Val-Tyr-AMC peptide substrate, 2–6 h, 12–16 h, and 2 days after injury. Twenty healthy children admitted for planned inguinal hernia repair served as controls.

**Results:**

Statistically significant elevation of plasma c-proteasome activity was noted in children with mild head injury 2–6 h, 12–16 h, and 2 days after the injury.

**Conclusions:**

Authors observed a statistically significant upward trend in the c-proteasome activity between 2–6 and 12–16 h after the mild head injury, consistent with the onset of the symptoms of cerebral concussion and a downward trend in the c-proteasome activity in the plasma of children with mild head injury between 12–16 h and on the second day after the injury, consistent with the resolving of the symptoms of cerebral concussion. Further studies are needed to demonstrate that the proteasome activity could be a prognostic factor, which can help in further diagnostic and therapeutic decisions in patients with head injury.

## Introduction

Head trauma occurs commonly in children. Most children with head trauma are young, male, and have a mild injury [31]. Mild head injury is generally associated with symptoms such as a brief loss of consciousness, disorientation, or vomiting. Patients with mild head injury usually have GCS scores of 13 to 15, measured approximately 30 min after the injury [30, 31]. The onset of impairment is rapid, but usually short-lived, and generally resolves spontaneously. Still, head injury is the most common cause of death and disability during childhood [19, 30, 31].

Consequently, children after mild head injuries are commonly admitted to emergency or surgical departments, overdiagnosed, and hospitalized. This large group of patients produces significant costs to the public health system. Therefore, new biochemical markers of mild head injuries are needed.

Ubiquitin-proteasome system is the principal enzyme system responsible for protein degradation, which has been reported to be abundantly expressed in the various cell types of the central nervous system [9, 28]. The addition of polyubiquitin chains to a protein results in its translocation to the proteasome, a large multi-subunit protease, which then results in its degradation. Proteasome catalytic function not only degrades most cytosolic proteins, but plays an essential role in numerous cellular processes including cell cycle programming, transcriptional control, signal transduction, and the elimination of damaged proteins [37]. The clearance of damaged proteins is indispensable in order to provide the necessary conditions for cellular repair and plasticity. Induction of proteasome has been reported after different traumatic stressors, such as hyperoxia, radiation, or oxidative damage [1, 32]. It is known that the oxidized proteins are tagged for degradation, and that activity of proteasome complex can be induced by oxidative protein damage in cell culture [32]. Recent in vitro study reports have demonstrated ubiquitin-proteasome enzyme system playing an essential role in rapid phase of ischemic preconditioning of cultured neurons [21, 22]. Also, ubiquitin-proteasome system activation is one of the important mechanisms regulating the cell death pathways in ischemic neurons [33]. Study by Szabo et al showed that experimental brain trauma elevates the levels of protein oxidation in the injured cerebral cortex. Correlation analysis showed that protein oxidation was positively associated with proteasome activity [32].

Moreover, patients with head injury exhibit negative nitrogen balance, enhanced rates of whole body protein breakdown, and a sustained rise in urinary 3-methylhistidine excretion, an index of muscle myofibrillar protein breakdown [18]. Overexpression of multiple components of the ubiquitin-proteasome proteolytic pathway in muscle biopsies from head trauma patients strongly suggests that this pathway is involved in the breakdown of contractile protein in humans [18].

The purpose of this novel human study was to characterize changes following mild head injury in children in the circulating 20S proteasome activity.

## Methods and materials

### Patients

The study was approved by the local Ethics Committee as an audit of a clinically agreed-upon protocol of investigation and treatment. All parents of the patients gave informed consent for both clinical and biochemical follow-up. The study population comprised 50 children admitted due to mild head injury to Department of Pediatric Surgery of Medical University of Bialystok, between 2010 and February 2014. Patients were randomly included in the study. Patients were aged 11 months to 17 years (median = 10.07 + −1.91 years). There were 19 girls and 31 boys. Falls were the most common mechanism of injury for our patients sustaining mild head injury, followed by motor vehicle crashes, pedestrian and bicycle accidents, and sports-related trauma. The diagnosis of mild head injury was based upon the following symptoms: loss of consciousness, amnesia, vomiting, severe headache, GCS score 13–15, and normal head CT. Patients with mild head injury were managed according to the conventional lines of head injury treatment in our department.

Exclusion criteria were GCS score below 13, abnormal head CT, hospital admission later than 6 h after injury, severe preexisting infections, and other diseases that required long-term medication.

Twenty healthy children aged 1–14 years (median = 4.36 + −2.03 years), admitted to the Department of Pediatric Surgery for planned inguinal hernia repairs between 2010 and 2012 (6 girls, 14 boys), served as controls. Exclusion criteria were hospital admission later than 6 h after injury, severe preexisting infections, and diseases that required long-term medication.

### Methods

Venous blood samples (1–2 ml) were drawn after admission, 2–6, and 12–16 h after the injury and in the morning of the subsequent 2 days along with the routine laboratory work-up. Blood samples were collected in plasma test tubes (EDTA tubes), plasma prepared according to the standard hospital procedures and stored at −80 °C until further analysis. After all blood samples were collected and patient data recorded, circulating 20S proteasome activity in the plasma was determined with the investigators blinded to the patient-related data.

The thawed plasma samples were centrifuged to remove fibrinogen, and then, each sample was diluted to the protein concentration of 5.0 mg/mL with 100 mmol/L Tris-HCl (pH 7.5) supplemented with inhibitors of other proteases: ethylene diamine tetraacetic acid (EDTA, 1 mmol/L), trans-epoxysuccinyl-leucylamide (4-guanidino)butane (E-64, 10 μmol/L), and pepstatin (1 μmol/L) (Sigma, USA). Total protein concentration in plasma samples was determined using the Bio-Rad assay reagent with bovine serum albumin as the standard.

The proteasome activity in the plasma was measured using the assay designed to measure chymotrypsin-like protease activity (ChT-L) of proteasomes using the fluorogenic peptide substrate the Suc-Leu-Leu-Val-Tyr-AMC in the presence of sodium dodecyl sulfate (SDS) as an artificial proteasome activator. The reaction mixture (total volume, 30 μL) contained 100 mmol/L Tris-HCl buffer (1 mmol/L EDTA, EGTA, 0.05 % SDS, pH 7.5), and 10 μL 0,5 mmol/L Suc-Leu-Leu-Val-Tyr-AMC substrate. Ten microliters of nonactivated plasma sample or 10 μL of plasma sample activated for 15 min at room temperature with 10 % SDS added to the reaction mixture. To confirm the specificity of the assay, the plasma was preincubated with the selective proteasome inhibitor epoxomicin (1.0 μmol/L) for 15 min before the addition of a substrate. The samples were incubated at 37 °C for 30–60 min, since during this time, a linear relation between time and product generation was obtained. After incubation, the reaction was stopped by the addition of 1 mL of 100 mmol/L monochloroacetate—30 mmol/L sodium acetate, and the samples were centrifuged to remove any insoluble material. Fluorescence of the released AMC was determined at 380 nm excitation and 460 nm emission wavelength in a Hitachi F-2000 fluorimeter. The 20S proteasome ChT-L activity was calculated from the differences between the fluorescence in the sample incubated with the substrate and the sample incubated with 0.1 % dimethyl sulfoxide (DMSO). One unit of the 20S proteasome ChT-L activity was expressed as the amount of AMC released from the substrate per minute (pmol/min). All assays were performed in triplicates.

For identification of 20S proteasome antigen, a plasma sample (20 μg of total protein per line) was electrophoresed by SDS-polyacrylamide gel electrophoresis (PAGE) in a 10 % separation gel under reducing conditions, and then proteins were electroblotted onto nitrocellulose membranes. The nitrocelluloses were incubated for 1 h with the monoclonal antibody reacting with the human β5 subunit (C2) of the 20S proteasome (Affiniti Research Products Ltd., UK). The immunoreactive proteins were visualized with an alkaline phosphate-conjugated anti-rabbit IgG as the secondary antibody and p-nitrophenyl phosphate as the phosphatase substrate (Sigma, USA).

### Statistics

Proteasome activity is described as median with 25th and 75th percentiles. All data are presented along with the exact number of measurements. Because the proteasome activity in the plasma of our patients did not pass the normality test, the Mann-Whitney *U* test and the Kruskal-Wallis *H* test were used to compare differences between groups. Statistical analyses were calculated with the STATISTICA PL release 10.0 Program. A two-tailed *p* < 0.05 was considered significant.

## Results

The circulating proteasome (c-proteasome) activity was detectable in all plasma specimens from patients and controls.

**Fig. 1:**
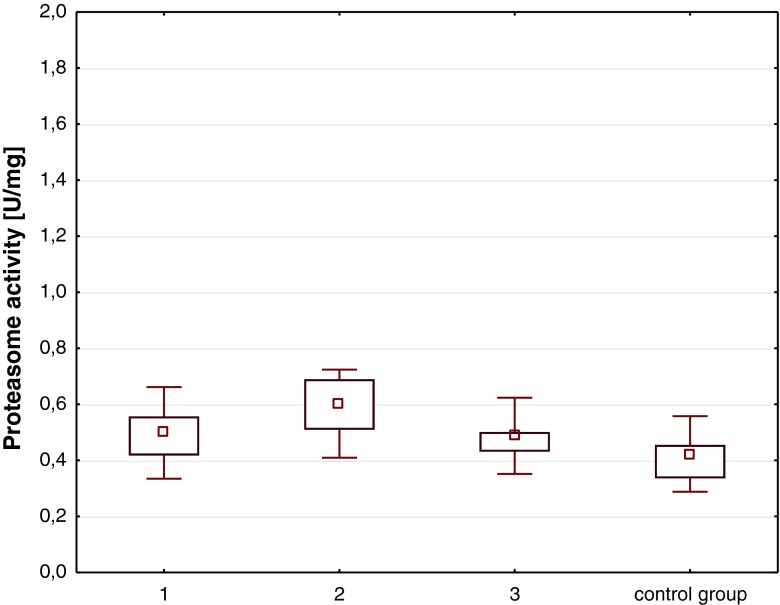
Circulating proteasome activity in the plasma of children, 2–6 h (1, *n* = 50), 12–16 h (2, *n* = 45), 2 days (3, *n* = 41) after mild head injury and in control group

In the group of our patients with mild head injury, the c-proteasome activity 2–6 h after the injury, 12–16 h after injury, and 2 days after injury were above the range of activity measured in controls which was statistically significant (Fig. 1). There was an upward trend in the c-proteasome activity between 2–6 and 12–16 h after the injury, consistent with the onset of the symptoms of cerebral concussion and a downward trend in the c-proteasome activity in the plasma of children with mild TBI between 12–16 h and on the second day after the injury, consistent with the resolving of the symptoms of cerebral concussion. The c-proteasome activity was not associated with age or sex (*p* > 0.05, data not shown).

## Discussion

Mild head injury usually occurs due to contact or acceleration-deceleration forces. The goal of the evaluation of children with head trauma is to identify those who may require immediate intervention or close follow-up. Although associated intracranial injury occurs in a small percentage of those children who have a normal neurologic examination at presentation, it must be recognized in order to prevent deterioration and subsequent morbidity or mortality [19, 30, 31].

Mild head injury causes a reduction in cognitive capacity [27, 29], possibly due to deterioration in the molecular substrates that support synaptic plasticity and function. Most forms of synaptic plasticity involve protein synthesis and limited protein degradation [11], which are under the spectrum of the action of proteasome complex. The proteasome system is responsible for the degradation of altered cellular proteins, including those modified by reactive oxygen species. Oxidized proteins are tagged for degradation either by the change of hydrophobicity or by ubiquitination, such that residual amino acids can be recycled for protein synthesis [6]. In our study, we wanted to check the hypothesis that c-proteasome activity in the plasma of children with minor head injury is associated with the onset and resolving of symptoms of cerebral concussion.

Several lines of evidence strongly suggest the ubiquitin-proteasome cascade is involved in responses to CNS stress or injury. Weih et al demonstrated that proteasome activation is needed for the proteolysis of oxidized proteins in cortical neurons under glucose and oxygen deprivation [34]. Another study showed that enhancement of proteasome activity potentially reduces neuronal vulnerability to oxidative injury [35]. A transgenic mouse study indicated that loss of an individual proteasome subunit—LMP2—alters mice brain function [20]. Using antibodies that recognize the entire 20S proteasome complex, Mengual et al [23] found that the proteasome is ubiquitously, although not homogeneously, expressed in rat CNS, and that subcellular distribution is localized to cytoplasmic, nuclear, dendritic, and axonal processes, and in synaptic boutons. Also in the group of our patients with mild head injury, the c-proteasome activity 2–6 h after the injury, 12–16 h after injury, and 2 days after injury were above the range of activity measured in controls which was statistically significant.

Earlier findings suggested that systemic 20S proteasome concentrations reflect cellular damage independent of the underlying cause of the disease and circulating proteasomes might be a useful biomarker to assess disease severity or progression [6, 10, 17]. In our study, we used assay of plasma 20S proteasome ChT-L activity for the measurement of circulating proteasomes. Several authors confirmed the specificity of this assay with highly selective proteasome inhibitors [25]. The most common method of detecting circulating proteasomes in plasma or serum is the enzyme-linked immunoabsorbent assay (ELISA) test employing antibodies directed against the constitutive α6 (C2) subunits of the 20S proteasome [2, 5]. ELISA-based assay does not provide information about circulating proteasome activity in the plasma, since only intact 20S proteasome complexes, but not subunit fragments, are enzymatically active. The method allows for the detection of only the intact 20S proteasome complexes, since free catalytic subunits are enzymatically inactive, so 20S proteasome ChT-L activity assay in plasma is a good alternative to ELISA for the measurement of circulating proteasomes [2, 5].

A decline in proteasome function is believed to underline many neurodegenerative diseases [12], where impaired function of the proteasomes has been proposed to contribute to the pathogenesis of degenerative diseases because of an accumulation of abnormal proteins [2, 7, 13, 14]. Impairment in the ubiquitin-proteasomal system also has been suggested as one of the underlying mechanisms of Parkinson’s and Alzheimer’s disease [3, 15]. Study of Alzheimer disease autopsy brain samples suggested that 19S and 20S proteasome subunits are localized in neurons [15].

In contrast to its role as pathogenic factor in neurodegenerative diseases, limitation of protein turnover after cellular damage would rather support regenerative processes. Increasing evidence indicates that reversible proteasome inhibitors can be therapeutic agents even neuroprotective during ischemic brain injury [36, 38]. Reduction of proteasome function is seen as key for reducing CNS damage from acute traumatic brain injury and stroke. Several studies have demonstrated that suppression of function by proteasome inhibitor administration leads to better outcome from CNS injury [8, 35, 38]. In our experiments, we observed an upward trend in the c-proteasome activity between 2–6 and 12–16 h after the injury, consistent with the onset of symptoms of cerebral concussion and a downward trend in the c-proteasome activity in the plasma of children with mild head injury between 12–16 h and 2 days after the injury, consistent with the resolving of the symptoms of cerebral concussion.

Another group of proteasome family—immunoproteasomes have been detected in human brains and indicate that immunoproteasomes participate in normal neuronal physiology [4, 24, 26]. A study of human brain injury by Majetschak et al, showed a progressive rise of CSF ubiquitin levels in traumatic brain injury patients who succumbed to their injuries compared with those who survived [16].

Our observations are the first to show an increase in circulating proteasome activity in the plasma in children hospitalized because of mild head injury. Our results also demonstrate, that molecular changes affecting protein metabolism, occur soon after head injury, and there is a downward trend in the c-proteasome activity in the plasma of children with mild head injury along with the resolving of the symptoms of the cerebral concussion.

## Conclusions

Authors observed a statistically significant upward trend in the c-proteasome activity between 2–6 and 12-16 after the mild head injury, consistent with the onset of the symptoms of cerebral concussion, and a downward trend in the c-proteasome activity in the plasma of children with mild head injury between 12–16 h and on the second day after the injury, consistent with the resolving of the symptoms of cerebral concussion. Further studies are needed to demonstrate that the proteasome activity could be a prognostic factor, which can help in further diagnostic and therapeutic decisions in patients with head injury.
